# Rush Medical College

**Published:** 1853-11

**Authors:** 


*Rush Medical College.*

THE introductory to the regular course of lectures in this institution was delivered on the 7th inst., by Professor Herrick.

The subject was the influence of climatic and other natural agenceis, in effecting change in man's physical and mental nature. The class in attendance is unusually large for the commencement of the session. The friends of the college will be gratified to know that its prospects were never more gratifying, than at present.

